# Effects of visualizing statistical information – an empirical study on tree diagrams and 2 × 2 tables

**DOI:** 10.3389/fpsyg.2015.01186

**Published:** 2015-08-26

**Authors:** Karin Binder, Stefan Krauss, Georg Bruckmaier

**Affiliations:** Mathematics Education, Faculty of Mathematics, University of RegensburgRegensburg, Germany

**Keywords:** Bayesian reasoning, 2 × 2 table, natural sampling tree, natural frequencies, visual representation

## Abstract

In their research articles, scholars often use 2 × 2 tables or tree diagrams including natural frequencies in order to illustrate Bayesian reasoning situations to their peers. Interestingly, the effect of these visualizations on participants’ performance has not been tested empirically so far (apart from explicit training studies). In the present article, we report on an empirical study (3 × 2 × 2 design) in which we systematically vary visualization (no visualization vs. 2 × 2 table vs. tree diagram) and information format (probabilities vs. natural frequencies) for two contexts (medical vs. economical context; not a factor of interest). Each of *N* = 259 participants (students of age 16–18) had to solve two typical Bayesian reasoning tasks (“mammography problem” and “economics problem”). The hypothesis is that 2 × 2 tables and tree diagrams – especially when natural frequencies are included – can foster insight into the notoriously difficult structure of Bayesian reasoning situations. In contrast to many other visualizations (e.g., icon arrays, Euler diagrams), 2 × 2 tables and tree diagrams have the advantage that they can be constructed easily. The implications of our findings for teaching Bayesian reasoning will be discussed.

## Introduction

Bayes’ formula is vitally important in many areas, such as in medicine or law. Unfortunately, both laymen and professionals have trouble understanding and combining statistical information effectively. The resulting misjudgments can have severe consequences, for example when juries must convict or acquit defendants based on probabilistic evidence in legal trials (Hoffrage et al., 2000; Krauss and Bruckmaier, 2014), or when physicians have to understand and to communicate what a positive test result really means, for example in a HIV or cancer test (Ellis et al., 2014). Consider, for instance, the classic mammography problem (adapted from Eddy, 1982; see also Gigerenzer and Hoffrage, 1995; Siegrist and Keller, 2011; Micallef et al., 2012; Garcia-Retamero and Hoffrage, 2013).

Mammography Problem (Probability Format):

The probability of breast cancer is 1% for a woman who participates in routine screening. If a woman who participates in routine screening has breast cancer, the probability is 80% that she will have a positive test result. If a woman who participates in routine screening does not have breast cancer, the probability is 9.6% that she will have a positive test result. What is the probability that a woman who participates in routine screening and receives a positive test result has breast cancer?

Answer: ______ %

According to Bayes’ theorem, the resulting posterior probability P(B|M+) is:

$$P(B|M+)=\frac{P(M+|B)\cdot P(B)}{P(M+|B)\cdot P(B)+P(M+|\;\neg B)\cdot P(\neg B)}\;\;\;\;\;\;\;\;\;\;\;\;\;\;\;\;\;\;\;\;\;\;\;\;\;\;\;\;\;\;\;\;=\frac{80\%\;\;\cdot \;1\%}{80\%\;\cdot \;1\%\;+\;9.6\%\;\cdot \;99\%}\approx 7.8\%$$

The correct result 7.8% is much lower than most people, including physicians, would expect (Eddy, 1982). Several studies show that medical doctors (Hoffrage and Gigerenzer, 1998; Garcia-Retamero and Hoffrage, 2013), patients (Garcia-Retamero and Hoffrage, 2013), legal professionals (Hoffrage et al., 2000), and students (Ellis et al., 2014) have difficulties with similar tasks. In order to help people to understand the situation, Gigerenzer and Hoffrage (1995) replaced the probabilities in Eddy’s task by natural frequencies.

Mammography Problem (Natural Frequency Format):

100 out of 10,000 women who participate in routine screening have breast cancer. Out of 100 women who participate in routine screening and have breast cancer, 80 will have a positive result. Out of 9,900 women who participate in routine screening and have no breast cancer, 950 will also have a positive result. How many of the women who participate in routine screening and receive a positive test result have breast cancer?

Answer: ____ out of ____

The percentage of correct responses increased from about 10–20% to about 50% in 15 different Bayesian reasoning tasks, including the mammography problem (Gigerenzer and Hoffrage, 1995). While the facilitating effect of natural frequencies is accepted by now, scholars differ in explaining this effect. Gigerenzer and Hoffrage (1995), for instance, argue that the human mind is evolutionarily adapted to the information format of natural frequencies (“ecological rationality”) that result from a natural sampling process (Kleiter, 1994). Other theorists, however, claim that essentially the partitive information structure is responsible for the facilitating effect (“nested sets hypothesis” e.g., Girotto and Gonzalez, 2001; Sloman et al., 2003; Barbey and Sloman, 2007). Some scholars suggest that two different cognitive systems (“dual process theory” Sloman, 1996; Kahneman and Frederick, 2005; Barbey and Sloman, 2007) may be responsible for inferences with respect to the different information formats. While probability format triggers intuitive thinking according to system 1 (“associative system” see also Sloman, 1996), which may lead to base rate neglect, natural frequency format triggers deliberate reasoning according to system 2 (“rule based system”). Advocates of the dual process theory often support the nested sets hypothesis (e. g., Barbey and Sloman, 2007). For a discussion of the concept of natural frequencies see Gigerenzer and Hoffrage (1999), Lewis and Keren (1999), Mellers and McGraw (1999), Girotto and Gonzalez (2001, 2002), Hoffrage et al. (2002), Barbey and Sloman (2007), or Sirota et al. (2015a).

In fact, there are recommendations that natural frequencies should become part of the training for all medical students (Gigerenzer, 2013) and, moreover, should be part of elementary school curricula (Gigerenzer, 2014). Although the effect of numerical format (probabilities vs. natural frequencies) is quite substantial, it has to be noted that there is still potential for improvement (“only” approximately 50% correct solutions).

**FIGURE 1:**
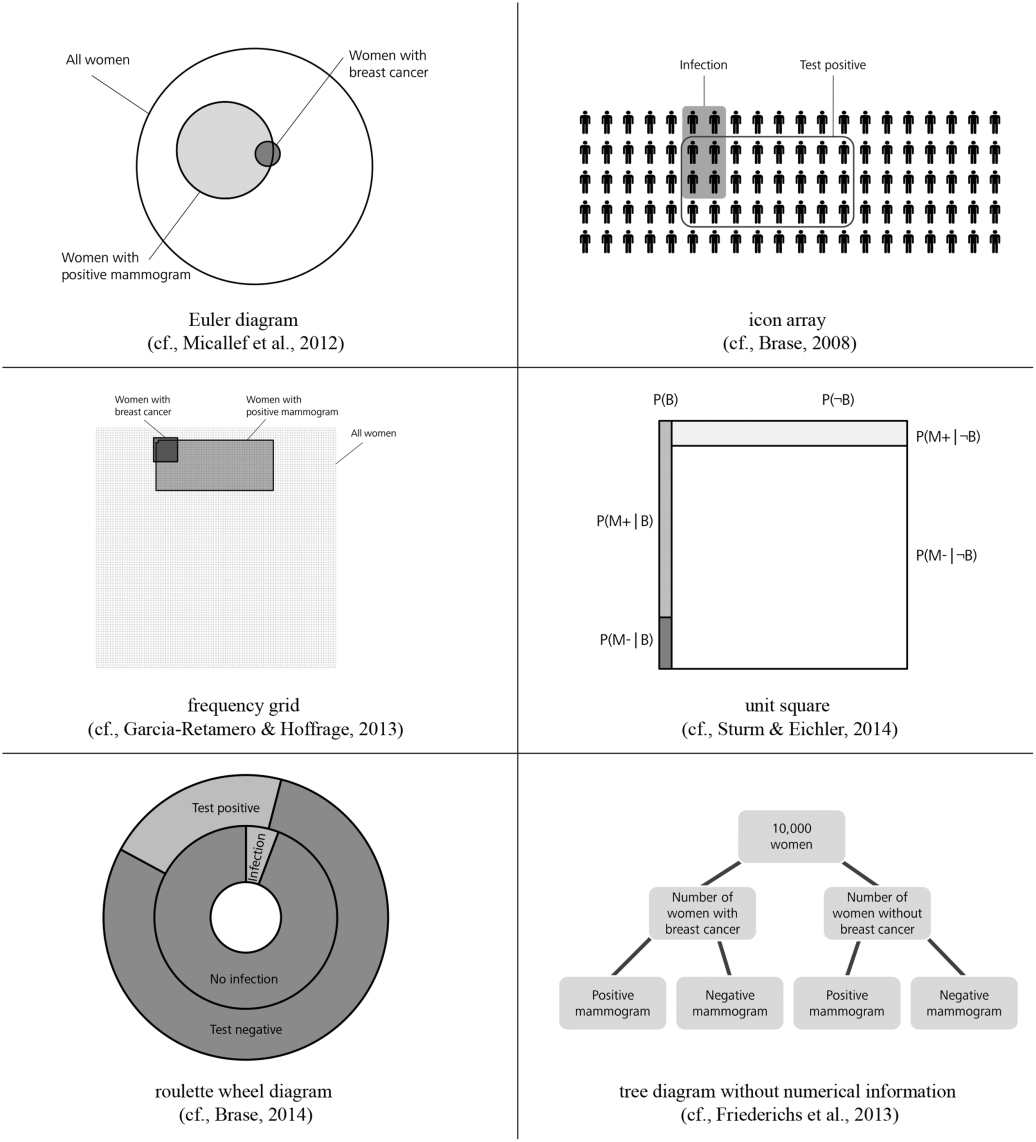
**Risk communication via Euler diagram, icon array, frequency grid, unit square, roulette wheel diagram, and tree diagram without numerical information**.

Another idea to improve insight into Bayesian reasoning situations is the additional representation of visual aids such as *Euler diagrams*, *icon arrays*, *frequency grids*, *unit squares*, *roulette wheel diagrams*, and *tree diagrams* (see **Figure 1**). According to the nested sets hypothesis, most of these visual aids represent the set-subset relation of the information. For an overview of possible visualizations see Paling (2003) or Spiegelhalter et al. (2011). **Figure 1** shows some visual aids which have been tested empirically so far.

Sloman et al. (2003), Brase (2008), Micallef et al. (2012), and Sirota et al. (2014b) investigated to what extent the presentation of *Euler diagrams* can boost performance in Bayesian reasoning tasks. They obtained different findings regarding the effectiveness of Euler diagrams, a result which potentially is affiliated to the various types of participants in their studies. *Icon arrays* (also called *pictographs*) are matrices of small figures that represent the given information. Within an array, some of the icons are shaped in a special form or are colored in order to show the number of figures that fulfill a special feature. Brase (2008, 2014) and Zikmund-Fisher et al. (2014) recommend risk communication via icon arrays since their studies showed a positive influence of this visual aid (for a discussion of the concept of “iconicity” see, e.g., Sirota et al., 2014b). *Frequency grids* are close to icon arrays showing the overall number of persons in a large grid where particular subsets of persons are marked characteristically. Garcia-Retamero and Hoffrage (2013) found that both doctors’ and patients’ performance increased when frequency grids are provided (see also Garcia-Retamero et al., 2015). *Unit squares* (Bea, 1995; Sturm and Eichler, 2014) also mirror the statistical information geometrically and represent the different sets of the task. Bea (1995) recommends the visualization of information via a unit square since his research reveals substantial improvement in performance. *Roulette wheel diagrams* (Brase, 2014) summarize the information presented by two circles (inner and outer circle) which represent different subsets of the problem. However, the additional representation of a roulette wheel diagram causes only a very small or even no improvement in performance compared to versions without any visual aid (Brase, 2014). Friederichs et al. (2014) investigated *tree diagrams* without numerical values (except an imaginary sample size). In their studies, performance in probability versions with tree diagrams was similar to the performance in natural frequency versions without visualization.

Note that one can differentiate between two types of studies in general: On the one hand there are training studies where participants are explicitly instructed in how to create visual aids on their own, and consequently, how to combine the given numbers to arrive at the solution. The effect of this “teaching” then is investigated by presenting additional problems without visualizations (e.g., Sedlmeier and Gigerenzer, 2001; Ruscio, 2003; Sirota et al., 2015b). On the other hand there are studies – as in our study – where word problems are accompanied by visualizations (e.g., Brase, 2008; Garcia-Retamero and Hoffrage, 2013). Note that in the latter studies, it is *not* taught how to construct visualizations for fostering insight, and therefore, there is no prior instruction as to how the given numbers should be applied to infer the solution. The visualizations in this case rather illustrate the information of the given problem in parallel.

Interestingly, the beneficial effect of 2 × 2 tables and tree diagrams presently was investigated only in the context of training studies (e.g., Sedlmeier and Gigerenzer, 2001). This is astonishing since scholars commonly use tree diagrams (Kleiter, 1994; Gigerenzer and Hoffrage, 1995; Mandel, 2014; Navarrete et al., 2014) and 2 × 2 tables (Goodie and Fantino, 1996; Dougherty et al., 1999; Fiedler et al., 2000) containing numerical values in their research papers to represent Bayesian reasoning situations to their colleagues.

**FIGURE 2:**
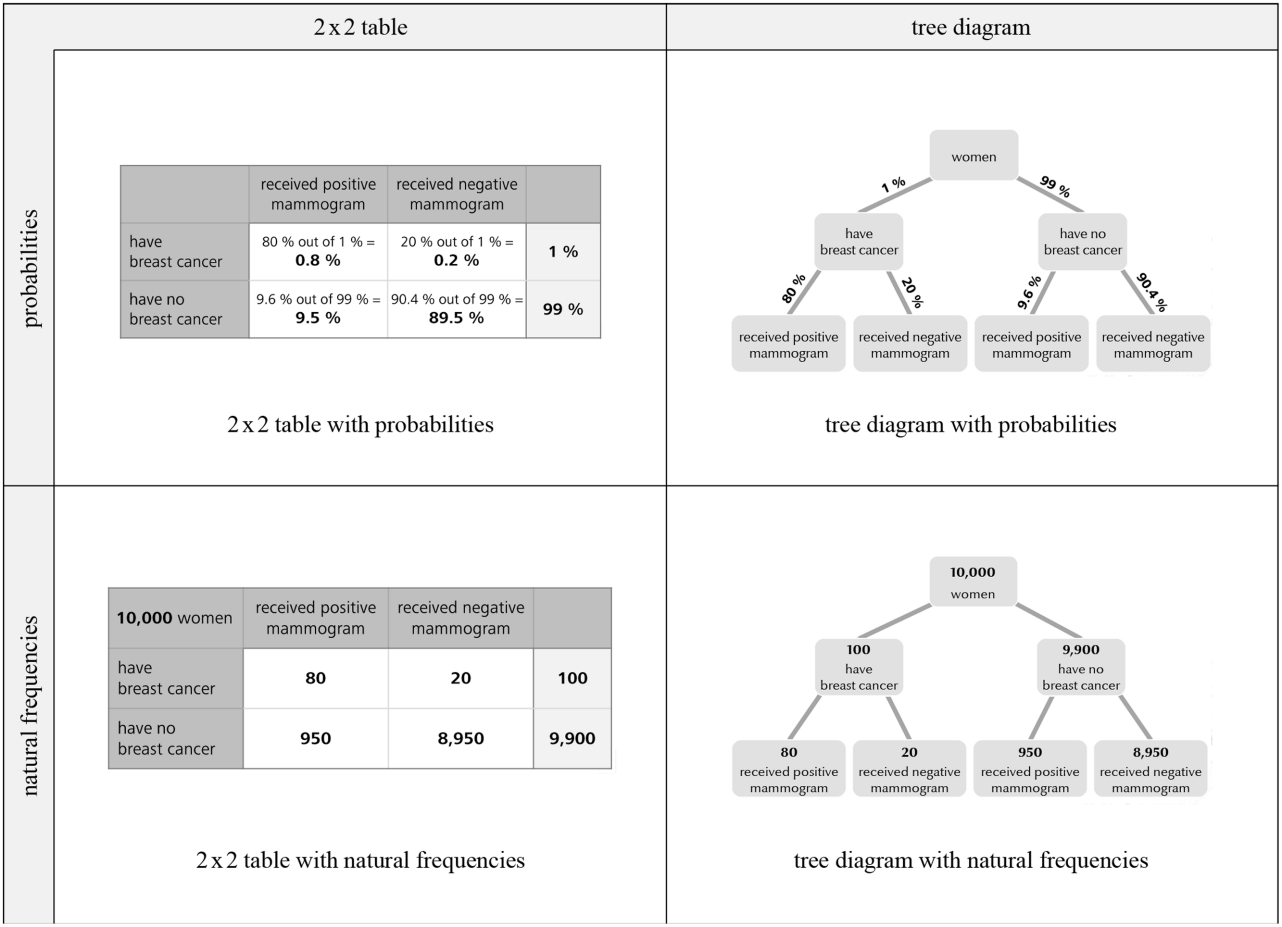
**Four resulting visualizations of the respective information format (mammography problem)**.

In the present paper we investigate how performance in Bayesian reasoning tasks can additionally be enhanced by providing 2 × 2 tables and tree diagrams containing numerical values. Since 2 × 2 tables and tree diagrams both can be equipped with natural frequencies or with probabilities we decided to test all four possible visualizations (compare **Figure 2**). Our hypotheses were:

• Hypothesis 1: Problems in which information is presented in natural frequencies are easier to solve than problems containing probabilities. This holds true when problems without visualization are compared (replication of previous studies) and when problems with visualizations are compared.
• Hypothesis 2: The additional presentation of visualizations of the numerical values (2 × 2 tables and tree diagrams) facilitates understanding. This holds for natural frequency and for probability versions as well.

We had no hypothesis as to which of both kinds of visualization is more beneficial. Furthermore we had no hypothesis on the effect of the problem context (we had chosen two problem contexts for mutual validation of our results; see **Table 1**).

**Table 1:** Design of the 12 tested problem versions.

		Context	
		Mammography problem	Economics problem
**Format**	**Probabilities**	• No visualization	• No visualization
		• 2 × 2 table	• 2 × 2 table
		• Tree diagram	• Tree diagram
				
	**Natural frequencies**	• No visualization	• No visualization
		• 2 × 2 table	• 2 × 2 table
		• Tree diagram	• Tree diagram

## Experimental Study

### Design

In a paper-and-pencil questionnaire participants were presented with two Bayesian reasoning tasks, the mammography problem and a short version of the economics problem (Ajzen, 1977; for problem formulations see **Table 2**). The design of the study includes two factors of interest (visualization and format of information) and one factor which was not of interest (context), resulting in a 3 × 2 × 2 design:

**Table 2:** Problem formulations.

	Mammography problem		Economics problem	
	Probability version	Natural frequency version	Probability version	Natural frequency version
**Cover story**	Imagine you are a reporter for a women’s magazine and you want to write an article about breast cancer. As a part of your research, you focuses on mammography as an indicator of breast cancer. You are especially interested in the question of what it means, when a woman has a positive result (which indicates breast cancer) in such a medical test. A physician explains the situation with the following information:		Imagine you are interested in the question, if career-oriented students are more likely to attend an economics course. Therefore the school psychological service evaluates the correlations of personality characteristics and choice of courses for you. The following information is available:	
				
**Version**	The probability of breast cancer is 1% for a woman who participates in routine screening. If a woman who participates in routine screening has breast cancer, the probability is 80% that she will have a positive test result. If a woman who participates in routine screening does not have breast cancer, the probability is 9.6% that she will have a positive test result.	100 out of 10,000 women who participate in routine screening have breast cancer. Out of 100 women who participate in routine screening and have breast cancer, 80 will have a positive result. Out of 9,900 women who participate in routine screening and have no breast cancer, 950 will also have a positive result.	The probability that a student attends the economics course is 32.5%. If a student attends the economics course, the probability that he is career oriented is 64%. If a student does not attend the economics course, the probability that he is still career-oriented is 60%.	325 out of 1,000 students attend the economics course. Out of 325 students who attend the economics course, 208 are career-oriented. Out of 675 students who not attend the economics course, 405 are still career-oriented.
				
**Visual aid**	• No visualization, or • 2 × 2 table (prob.), or • Tree diagram (prob.)	• No visualization, or • 2 × 2 table (nat. freq.), or • Tree diagram (nat. freq.)	• No visualization, or • 2 × 2 table (prob.), or • Tree diagram (prob.)	• No visualization, or • 2 × 2 table (nat. freq.), or • Tree diagram (nat. freq.)
				
**Question**	What is the probability that a woman who participates in routine screening and receives a positive test result has breast cancer?	How many of the women who participate in routine screening and receive a positive test result have breast cancer?	What is the probability that a student attends the economics course if he is career-oriented?	How many of the students who are career-oriented attend the economics course?
	Answer: _______ %	Answer: ____ out of ____	Answer: _______ %	Answer: ____ out of ____

• *Visualization:* no visualization vs. 2 × 2 table vs. tree diagram.
• *Format of statistical information:* probabilities vs. natural frequencies.
*Context:* mammography problem vs. economics problem (not a factor of interest).

Each participant received one of the two problem contexts with probabilities and the other problem with natural frequencies. Thereby the order of context and information format was varied systematically. Furthermore, if in one of the two problems, for instance, a 2 × 2 table was added, in the other problem either no visualization or a tree diagram was presented. There were no time constraints for completing the questionnaire (participants required about 20 min for both tasks). In **Table 1** the design, resulting in 12 tested versions, is illustrated, whereas in **Table 2** the corresponding problem formulations are denoted.

The key factor under investigation in the present article is the effect of visualization. Note that in contrast to most visual aids tested so far (**Figure 1**) our visualizations explicitly contain numerical information. It is generally possible to equip both 2 × 2 tables and tree diagrams with natural frequencies or with probabilities, respectively (**Figure 2**). The construction rationale for the visualizations was to provide statistical information that is also reported in the typical problem formulations. However, to “complete” the tree diagrams some information must be added that is not mentioned in the problem formulation (the information “20%” and “90.4%” in the probability tree or “20” and “8,950” in the frequency tree, respectively). In order to mirror these numerical values in the 2 × 2 table containing natural frequencies, one (of two possible) marginal distribution has to be depicted (**Figure 2**). Most problematic is the construction of the 2 × 2 table with probabilities. Such 2 × 2 tables usually contain conjoint probabilities, whereas Bayesian reasoning tasks contain conditional probabilities. The underlying relationship between both kinds of probabilities is included in the cells of the 2 × 2 tables (probabilities). It has to be noted that the 2 × 2 table (with conjoint probabilities), the 2 × 2 table (with natural frequencies) and the tree diagram (with probabilities) are part of the German school curriculum, whereas the tree diagram with natural frequencies (“natural frequency tree”)is not.

### Instrument

Each participant was presented two successive tasks which varied in terms of (1) visualization (no visualization vs. 2 × 2 table vs. tree diagram), (2) information format (probabilities vs. frequencies), and (3) problem context (mammography vs. economics problem). All versions begin with a cover story (see also **Table 2**); after that, one of three different kinds of visualization (including no visualization) was given (**Figure 2**). Finally, the question was provided in the same format as the information in the text.

The correct solution for the mammography problem is 80 out of 1,030 (about 7.8%), and for the economics problem 208 out of 613 (33.9%). Note that the corresponding algorithm to calculate the Bayesian posterior probability is identical for 2 × 2 tables concerning both information formats. However, the algorithm for computing P(B|M+) based on a tree diagram differs substantially with respect to both information formats.

A response has been classified as a correct “Bayesian answer” if the exact probability or frequency solution was provided, or the probability solution was rounded up or down to the next full percentage point (e.g., in the mammography problem the correct solution is 7.8%, therefore answers between 7 and 8% were classified as a correct solution; see also Gigerenzer and Hoffrage, 1995).

### Participants

The participants were *N* = 259 German secondary school students age 16–18. Students were recruited from 12 different classes (grade 11) at two Bavarian Gymnasiums. Note that in Germany there are different kinds of secondary school tracks. In order to study at a university, the Gymnasium (academic track) must be pursued. All students were familiar with 2 × 2 tables and tree diagrams containing probabilities and with 2 × 2 tables containing frequencies but not with natural frequency trees.

The study was carried out in accordance with the University Research Ethics Standards. The principals of both schools approved conduction of the study (this is mandatory in Germany when testing school students). When conducting the study we did not collect personal data (our questionare did not include questions with regard to age, gender etc.). Students were informed that their participation was voluntary (two students refrained from participating) and anonymity was guaranteed. After the study participants were debriefed.

## Results

**FIGURE 3:**
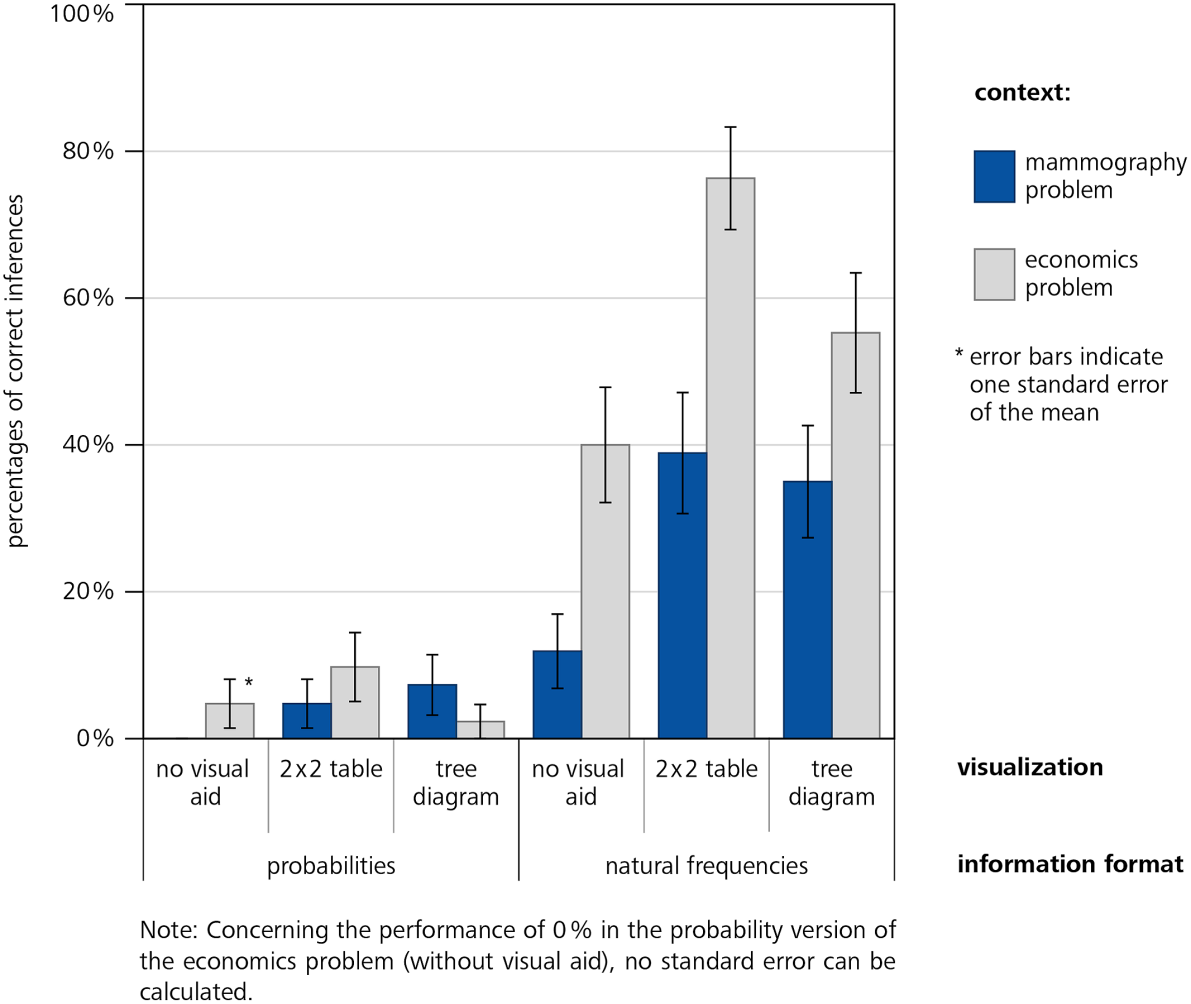
**Participants performance (error bars indicate the SE)**.

Our study showed three important findings (**Figure 3**). First, students’ performance was higher when information in the problems was presented in natural frequencies (42% correct inferences, averaged across context and visualization) instead of probabilities (5%), which supports our hypothesis 1. This finding holds when only problems *without* visualizations are compared (26% correct inferences in natural frequency versions vs. 2% correct inferences in probability versions, averaged across both contexts, which replicates previous findings, e.g., Gigerenzer and Hoffrage, 1995; Siegrist and Keller, 2011) and when problems *with* visualizations are compared (51% correct inferences in natural frequency versions vs. 6% correct inferences in probability versions, averaged across both contexts).

Second, the additional presentation of visualizations helps understanding (hypothesis 2): Averaging across all versions *with* visualization yields higher performance (28%) than averaging across all versions *without* visualizations (14%). Note that this difference is much stronger in the natural frequency versions (51% vs. 26%, averaged across both contexts) than in the probability versions (6% vs. 2%, see **Figure 2**). The fact that probability visualizations only have very limited effect is irritating since these visual aids are frequently applied in statistical text books (see Discussion).

Furthermore, participants showed better performance in almost every version of the economics problem (30% correct inferences, averaged across format of information and visualization) compared to the respective versions of the mammography problem (16%). Possible reasons will be debated in Section “Discussion.”

In order to analyze the impact of information format and visualization simultaneously we ran binary logistic regressions. Since we had no hypothesis on possible effects of problem context we performed two logistic regressions for the mammography problem and for the economics problem separately. The independent variables were visualization (only distinguishing between *no visualization* vs. *visualization*) and information format, respectively. The dependent variable was the correctness of the solution (1 – correct solution, 0 – incorrect solution). The results of the statistical analyses are illustrated in **Table 3**. For both contexts model 1 shows the impact of information format, whereas model 2 shows the impact of information format and visualization simultaneously.

**Table 3:** Results of binary logistic regression; independent variables: visualization and information format; dependent variable: correctness of solution.

	Dependent variable: correctness of solution			
	Mammography problem		Economics problem	
	Model 1	Model 2	Model 1	Model 2
Independent variable	EXP(B)	EXP(B)	EXP(B)	EXP(B)
Format of information	9.40^∗∗∗^	10.44^∗∗∗^	22.44^∗∗∗^	24.73^∗∗∗^
Visualization		4.99^∗∗^		2.53^∗^
*R*^2^	0.19	0.27	0.41	0.44

*EXP(B): Odds ratio (indicates how many times the odds of solving the task is higher when the independent variable is 1, as compared to the independent variable of 0); R^2^: Goodness of fit (according to Nagelkerke).*

*^∗^significant at p = 0.05; ^∗∗^significant at p = 0.01; ^∗∗∗^significant at p = 0.001.*

In both problem contexts we found significant coefficients regarding information format (hypothesis 1) and visualization (vs. no visualization; hypothesis 2). Additional analyses revealed no statistical differences between 2 × 2 table and tree diagram in each information format. Although **Figure 3** suggests a possible interaction of format and visualization the regression does not yield a respective significant coefficient. Note that the seeming interaction between format and visualization may be due to the floor effect with respect to the probability versions. However, considering **Figure 2** it becomes clear that visualizations of the numerical values in probability versions do not help substantially.

## Discussion

According to general theories of information encoding and processing (e.g., Cognitive Load Theory, Sweller, 2003; Cognitive Theory of Multimedia Learning, Mayer, 2005), understanding of statistical information could be supported by presenting additional visual aids. In our study, participants’ performance in two Bayesian reasoning tasks was higher when additionally 2 × 2 tables and tree diagrams containing natural frequencies were presented. However, when applying these visual aids for Bayesian inferences, the information format should be taken into account: both tools have only very limited effects when probabilities are included. Since in statistics text books and school curricula both probability visualizations – but not frequency trees – commonly are applied in order to foster insight, this finding is quite remarkable.

In general, our results are in line with the “frequentist hypothesis” (Gigerenzer and Hoffrage, 1995; Cosmides and Tooby, 1996) as well as the “nested sets hypothesis” (Barbey and Sloman, 2007). Regarding all problem versions, natural frequency versions resulted in higher performance levels compared to the respective probability versions. The low performance, however, in the natural frequency version of the mammography problem without visualization indicates only moderate statistical literacy in the participants of our study. Interestingly, the performance in the economics problem was much better than in the mammography problem under almost every condition. A possible reason might be the extreme base rate (1%) in the mammography problem which basically constitutes the cognitive illusion (in contrast, the result of the economics problem is no longer counterintuitive). Another reason might be that the context of the economics problem is more adapted to the living environment of young people (a strong dependency from the problem context was also found by Siegrist and Keller, 2011). The more complicated terminology or taxing cognitive capacity in the mammography problem could also account for the deviant effects in the different contexts (e.g., Lesage et al., 2013; Sirota et al., 2014a).

The need for tools for teaching statistics is repeatedly stressed (Gigerenzer, 2013, 2014; Navarrete et al., 2014). There are several teaching studies (Sedlmeier and Gigerenzer, 2001; Wassner, 2004; Mandel, 2015; Sirota et al., 2015b) where the solution process of a Bayesian reasoning problem is explained explicitly, e.g., with the help of visualizations, and the effect of teaching is investigated. For instance, it is even possible to advise students to imagine an arbitrary sample when given a probability version and then to construct a frequency table or tree diagram accordingly (by increasing the size of the arbitrary sample whole numbers always can be reached for each respective subset). Furthermore Hoffrage et al. (submitted, same issue) instructed participants to solve complex Bayesian reasoning problems (e.g., with more than one cue) by translating the given information in terms of probabilities into natural frequencies and to construct a corresponding tree diagram accordingly. Note again, that our study is not an explicit teaching study; nevertheless our findings have pragmatic implications for teaching Bayesian reasoning. Our visualizations have the advantage that they can be constructed easily by teachers or students. In contrast, the diagrams in **Figure 1** are complicated to produce, which is especially problematic when base rates are extreme. In the unit square, for instance, areas can become very small (in **Figure 1** therefore a higher base rate of the disease was chosen). Similarly, concerning the icon array, more symbols would be required in the case of small or unmanageable proportions (such as 1.25 or 9.6%) thus entailing an enormous effort. Our frequency visualizations, which of course can be combined with other visualizations (for an integration of a natural frequency tree and an icon array see, e.g., Mossburger, unpublished manuscript), thus may be a helpful aid for fostering statistical understanding and for teaching statistics in schools.

Note that 2 × 2 tables and tree diagrams containing natural frequencies can not only aid in Bayesian reasoning problems, but can also illustrate situations with two dichotomous features in general. For instance, it is possible to justify and explain the rules for multiplication and addition of conditional probabilities with natural frequency trees very easily (Mossburger, unpublished manuscript). Since 2 × 2 tables and tree diagrams containing natural frequencies can be provided long before students have to solve Bayesian reasoning problems, these visual aids offer the opportunity to consider various types of problems over a long period of a school or university curriculum.

## Conflict of Interest Statement

The authors declare that the research was conducted in the absence of any commercial or financial relationships that could be construed as a potential conflict of interest.
